# Molecular Basis of Artemisinin Derivatives Inhibition of Myeloid Differentiation Protein 2 by Combined in Silico and Experimental Study

**DOI:** 10.3390/molecules26185698

**Published:** 2021-09-20

**Authors:** Sennan Qiao, Hansi Zhang, Fei Sun, Zhenyan Jiang

**Affiliations:** 1 School of Pharmaceutical Sciences, Jilin University, Changchun 130021, China; qiaosn19@mails.jlu.edu.cn; 2 College of Basic Medical Sciences, Jilin University, Changchun 130021, China; zhanghansi@jlu.edu.cn

**Keywords:** molecular dynamics simulation, myeloid differentiation factor 2, artemisinin, anti-inflammatory, binding free energy

## Abstract

Artemisinin (also known as Qinghaosu), an active component of the Qinghao extract, is widely used as antimalarial drug. Previous studies reveal that artemisinin and its derivatives also have effective anti-inflammatory and immunomodulatory properties, but the direct molecular target remains unknown. Recently, several reports mentioned that myeloid differentiation factor 2 (MD-2, also known as lymphocyte antigen 96) may be the endogenous target of artemisinin in the inhibition of lipopolysaccharide signaling. However, the exact interaction between artemisinin and MD-2 is still not fully understood. Here, experimental and computational methods were employed to elucidate the relationship between the artemisinin and its inhibition mechanism. Experimental results showed that artemether exhibit higher anti-inflammatory activity performance than artemisinin and artesunate. Molecular docking results showed that artemisinin, artesunate, and artemether had similar binding poses, and all complexes remained stable throughout the whole molecular dynamics simulations, whereas the binding of artemisinin and its derivatives to MD-2 decreased the TLR4(Toll-Like Receptor 4)/MD-2 stability. Moreover, artemether exhibited lower binding energy as compared to artemisinin and artesunate, which is in good agreement with the experimental results. Leu61, Leu78, and Ile117 are indeed key residues that contribute to the binding free energy. Binding free energy analysis further confirmed that hydrophobic interactions were critical to maintain the binding mode of artemisinin and its derivatives with MD-2.

## 1. Introduction

Toll-like receptor 4 (TLR4), as a transmembrane protein, is one of the most studied pathogen-associated molecular patterns (PAMPs) recognition receptor of the Toll-like receptor family (TLRs), and plays an important role in innate and adaptive immune responses [1,2]. Abnormal activation of the TLR4 signaling pathway may lead to a broad variety of diseases, including allergic diseases, autoimmune disorders, infectious diseases, cardiovascular disease, obesity-associated metabolic diseases, neuronal degeneration and inflammatory bowel diseases [3,4,5,6,7,8,9]. Therefore, understanding the mechanism and function of TLR4 has attracted extensive attention, and can lay a foundation for the diagnosis and treatment of these diseases.

Previous study showed that TLR4 can be specifically recognized and activated by bacterial lipopolysaccharide (LPS) in association with the accessory proteins MD-2, leading to dimerization of TLR4-MD-2-LPS complex and to the release nitric oxide (NO) and pro-inflammatory cytokines such as tumor necrosis factor-α (TNF-α), interleukin-6 (IL-6), and interleukin-1β (IL-1β) [10,11,12,13,14]. Furthermore, TLR4 is insufficient for LPS recognition, and the physical association of an extracellular domain of TLR4 with MD-2 is a prerequisite for ligand-induced activation [10]. Crystallographic and NMR studies revealed that LPS binds to the hydrophobic pocket of MD-2, which is composed of a β-cup fold structure. Therefore, exploring small molecules that can competitively bind in the LPS-binding pocket of MD-2 was proposed as a way of inhibiting sustained activation and overactivation of the TLR4 signaling pathway.

Recent evidence suggests that artemisinin, a natural product derived from qinghao, has an anti-inflammatory activity that is linked to MD-2 inhibition [15,16,17]. Artemisinin has been widely used as a first-line antimalarial drug for the treatment of malaria [18]. Additionally, artemisinin has well-known antibacterial, antifungal, antileishmanial, antioxidant, antitumor, and anti-inflammatory activities [19,20]. Recently, Zhang et al. evaluated artemisinin as an anti-inflammatory agent in LPS-stimulated macrophages and mouse models [15]. Artemisinin and its derivatives showed similar potencies in inhibiting TLR4 signaling, whereas, the molecular mechanisms of interactions between artemisinin derivative and MD-2 remains unclear. The relationship between the interactions and the bioactivity of MD-2 is still elusive.

In the present study, the anti-inflammatory effect and the mechanism of artemisinin derivatives on MD-2 were investigated. First, we evaluated and compared the inhibitory effects of artemisinin and its derivatives on LPS-induced inflammation in BV2 cells. We found that artemether exhibits better anti-inflammatory activity performance than artemisinin and artesunate. Subsequently, molecular docking and molecular dynamics simulations were carried out in explicit water of 200 ns in length for different systems (Apo-MD-2, MD-2-Artemisinin, MD-2-Artemether, MD-2-Artesunate) to explore their detailed interactions at the molecular level. Here, we report the molecular mechanism between MD-2 and inhibitors, which may provide a new idea for developing new MD-2-targeted inhibitors.

## 2. Materials and Methods

### 2.1. *In Vitro Studies*

#### 2.1.1. Chemicals

Atremisinin, artesunate, and artemether (purity ≥ 98%) were purchased from Aladdin (Shanghai, China).

#### 2.1.2. BV2 Cell Culture

BV2 murine microglia were cultured in DMEM including 10% fetal bovine serum (FBS), 50 U/mL penicillin, and 50 μg/mL streptomycin at 37 °C in a 5% CO${}_{2}$ incubator. BV2 microglial cells were detached from the flask by a cell lifter when confluence was reached. The cells were seeded at a density of $4*10^{4}$ cells per well in 96-well plates. After overnight incubation, the medium was aspirated and changed to DMEM without FBS. The cells were then treated with 200 ng/mL of *E. coli* LPS and various concentrations (0.01, 0.1, 1, 10, 100 μM) of artemisinin, artesunate and artemether. After 24 h, the supernatant was collected for NO analysis and TNF-α ELISA. Meanwhile, the cells were stained by using CCK-8 for viability analysis or collected for IL-1β ELISA.

#### 2.1.3. NO Assay

A 100 μL portion of the supernatant medium was collected after the cells were treated for 24 h. The levels of nitric oxide (NO) in the culture media were measured using a nitric oxide assay kit (Beyotime) according to the manufacturer’s instructions. Absorbance was measured at 540 nm in a microplate reader (Infinite F50, Tecan, Zürich, Switzerland).

#### 2.1.4. Cell Viability Assay

Cell viability was assessed using a Cell Counting Kit 8 (CCK-8) assay. The cells were seeded at a density of $2*10^{4}$ cells per well in 96-well plates. After treatment with 200 ng/mL of *E. coli* LPS and various concentrations (0.01, 0.1, 1, 10, 100 μM) of artemisinin, artesunate, and artemether for 24 h, 100 μl/mL of the CCK-8 solution (Beyotime) was added to the cells and incubated at 37 °C for 1 h. Absorbance was measured at 450 nm in a microplate reader (Infinite F50, Tecan).

#### 2.1.5. ELISA Assays for TNF-α and IL-1β ELISA Assays

The TNF-α and IL-1β proteins were measured using commercially available ELISA kits (Boster Biological Technology co.ltd) according to the manufacturer’s instructions.

### 2.2. *In Silico Studies*

#### 2.2.1. Molecular Docking

**Figure 1:**
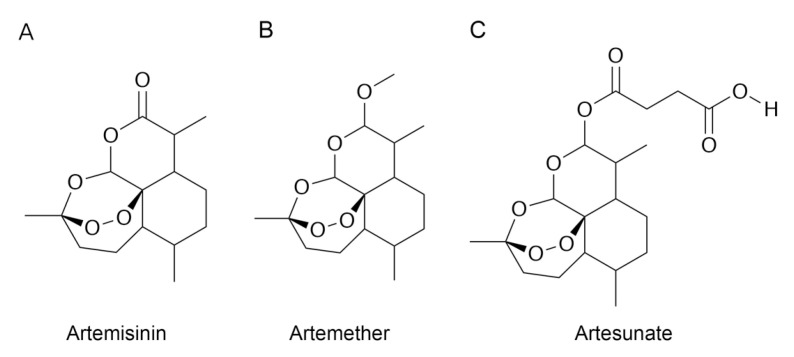
Chemical structures of (**A**) artemisinin, (**B**) Artemether, and (**C**) Artesunate.

The initial protein structure of MD-2 was obtained from RSCB Protein Data Bank (PDB ID: 3FXI) by removal of the TLR4 and original ligands [21]. Ligand structures of artemisinin, artesunate, and artemether were downloaded from the PubChem database (Figure 1). Molecular docking was performed for MD-2 and each ligand by using Autodock 4.2.6 [22]. Kollman united atom partial charges and only polar hydrogen atoms were taken into count in protein preparation. A grid box of 51 Å × 48 Å × 56 Å in the x, y, and z directions was centered at (30, −1.5, 18) Å of MD-2 structure with a spacing of 0.375 Å, and the size of grid box was determined to sufficiently cover the ligand binding pocket. Empirical scoring function and Lamarckian genetic algorithm were employed for docking simulations. All other docking parameters were set to their default values.

#### 2.2.2. Molecular Dynamics Simulation

To perform MD simulation, the best docking pose of a ligand in complex with lowest energy were chosen as the initial structure. The atomic partial charges of ligands were calculated using Gaussian 09 program at the B3LYP/6-31G* level followed by restrained electrostatic potential (RESP) fitting with Antechamber tool [23,24]. The topology of ligands in GMX format were obtained using ACPYPE program [25]. Amber99SB-ILDN force field implemented in GROMACS was used for molecular dynamics simulations [26,27]. Each complex (MD-2-Artemisinin, MD-2-Artesunate, MD-2-Artemether) and Apo-MD-2 with the default protonation states were automatically recognized by pdb2gmx in GROMACS. All systems were solvated in the TIP3P water model in a dodecahedron box, with the buffering distance set to 1.2 nm [28]. All systems were neutralized with the addition of NA${}^{+}$ and Cl${}^{-}$ ions in 150 mM concentration. For each system, energy minimization with a maximum of 5,000 steps was carried out using the steepest descent algorithm. Then, two 100 ps position-restrained simulations in NVT and NPT ensembles with harmonic constraints on all heavy atoms of system (force constant k = 1000 kJ · mol${}^{-1}\cdot$ nm${}^{-2}$) were used to equilibrate the system. Finally, a subsequent MD simulation of 200 ns length without any restraints was performed to study the equilibrium properties of each system. The v-rescale thermostat was applied for temperature coupling at 310 K with constant $\tau_{T}$ = 0.1 ps, while the Parrinello-Rahman barostat was applied for constant pressure control with $\tau_{P}$ = 4.0 ps and a compressibility of 4.5 × 10${}^{-5}$ bar${}^{-1}$ [29]. Long-range electrostatic interactions were computed using the Particle Mesh Ewald (PME) method [30]. The Coulomb and Lennard–Jones interactions were calculated using a cutoff of 1.2 nm. The integration time step was set to 2 fs and all hydrogen atoms were constrained using the LINCS algorithm [31].

#### 2.2.3. Binding Free Energy Calculations

The binding free energy of artemisinin, artesunate, and artemether binding to MD-2 was calculated using the Molecular Mechanics Poisson-Boltzmann Surface Area(MM/PBSA) method with the *g_mmpbsa* program for 200 snapshots from the last 20 ns of each simulations [32]. Both molecular mechanics potential energy (electrostatic + van der Waals interaction) and solvation free energy (polar + non-polar solvation energy) were calculated using default parameters. The binding energy was calculated as the sum of the electrostatic energy, the vander Walls energy, the polar solvation energy, and the non-polar solvation energy. All visualizations of the protein ligand complexes were performed using VMD software.

## 3. Results and Discussion

### 3.1. Cytotoxicity of Artemisinin Derivatives on the Viability of Cells

**Figure 2:**
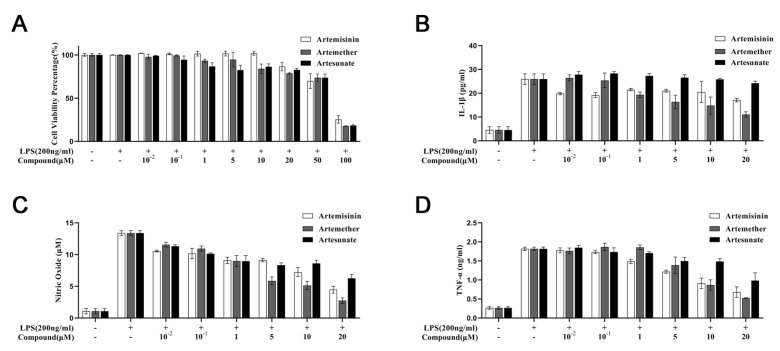
Effects of artemisinin, artesunate, and artemether on (**A**) cell viability, (**B**) interleukin-1β (IL-1β), (**C**) nitric oxide, and (**D**) tumor necrosis factor-α (TNF-α) production in lipopolysaccharide (LPS) treated BV2 microglial cells.

The effect of artemisinin, artemether, and artesunate on the viability of BV2 microglial cells was determined using a CCK-8 assay. As expected, the presence of LPS had no impact on the cell viability (Figure 2A). Cells exposed to varying concentrations (0.01–20 μM) of artemisinin and its derivatives with 200 ng/mL of LPS for 24 h also showed no significant cytotoxicity. However, when the cells were treated with artemisinin and its derivatives at concentrations higher than 50 μM with 200 ng/mL LPS, the cell viability was significantly decreased in all groups. Therefore, all subsequent experiments were performed at ligand concentrations (0.01–20 μM).

### 3.2. Effect of Artemisinin Derivatives on Levels of IL-1β, Nitric Oxide, and TNF-α

Next, to further examine whether artemisinin and its derivatives inhibited LPS-induced TLR4 signaling, we investigated the anti-inflammatory activity of artemisinin, artemether and artesunate. TLR4 is primarily expressed on microglia cells. Thus the inflammatory response of BV2 microglial cells can be induced by LPS treatment. During inflammation, NO and pro-inflammatory cytokines, including TNF-α and IL-1β are produced. As shown in Figure 2B–D, the levels of NO, TNF-α, and IL-1β were dramatically increased in response to LPS compared with the control group without LPS stimulation. This effect was significantly attenuated by pretreatment with the artemisinin derivatives compared with the LPS-induced group. Meanwhile, artemisinin, artemether, and artesunate inhibited LPS-induced TLR4 signaling of NO with IC${}_{50}$ values of 12.78 μM, 44.78 μM and 2.79 μM, respectively. These results indicate that artemisinin, artemether, and artesunate could inhibit LPS-induced TLR4 signaling, which is consistent with the results of a previous study [15]. Moreover, artemether exhibited a better anti-inflammatory activity performance over artemisinin and artesunate.

### 3.3. Molecular Docking

**Figure 3:**
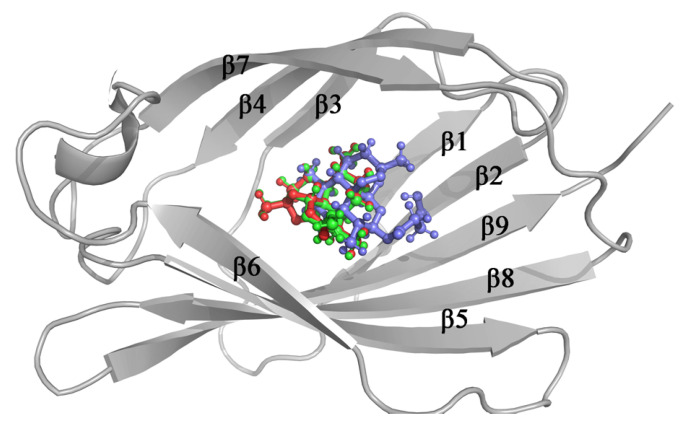
Structure of docked ligands at the binding site of MD-2, artemisinin (red stick), artemether (green stick) and artesunate (blue stick).

In order to investigate the interactions between each ligand and MD-2, molecular docking was performed to predict the binding mode between each ligand and MD-2 at the atomic level. As shown in Figure 3, MD-2 has a total of 9 β-strands and all three ligands are bound in the cavity. Artemisinin, Artesunate and Artemether have similar binding poses, with lowest binding energies of −28.28 kJ · mol ${}^{-1}$, −27.53 kJ · mol ${}^{-1}$, −26.74 kJ · mol ${}^{-1}$, respectively. Hydrophobic interactions have been observed between ligands and MD-2. The interacting residues are shown in Figure S1. Most of the residues were hydrophobic (residue Ile46, Leu61, Phe76, Leu78, Val135, and Phe151). It is reported that the residue Cys133 would stabilize the chemicals in the MD-2 binding cavity through covalent bond [33], while covalent interactions between the residue Cys133 and ligands in the MD-2 pocket were not observed. The binding pose for each MD-2 inhibitor in the MD-2 with the lowest energy was selected for molecular dynamics simulation.

### 3.4. Stability of MD-2-Ligand Complex

**Figure 4:**
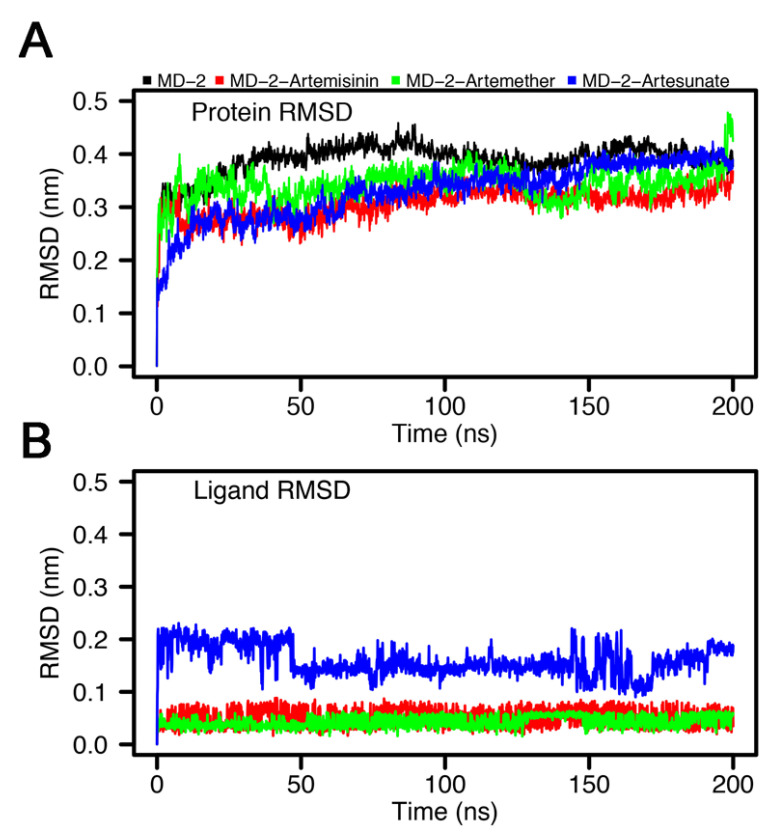
Root mean square deviations of (**A**) the MD-2 backbone atoms and (**B**) the ligand over 200 ns of simulations.

To further inspect the structural changes of MD-2 upon binding with different inhibitors, molecular dynamics simulations were performed using the docking complex and ligand-free MD-2 as the starting conformations. The root mean square deviation (RMSD) was calculated to reveal the stability for all systems. As shown in Figure 4A, the RMSD increases rapidly from the 0 nm and then reaches an equilibrium state after 50 ns in all systems. In the case of Apo-MD-2, the system remained stable around 0.399 nm, and ligand binding slightly decreased the RMSD values of MD-2 at 0.388 ± 0.010 nm, 0.360 ± 0.027 nm, and 0.324 ± 0.016 nm for artesunate, artemetherm, and artemisinin, respectively. The RMSD variations of all systems of protein backbond atoms at a 200 ns simulation time indicate that MD-2 in different systems are stable. Figure 4B shows the RMSD of all ligands during the simulations. The RMSDs of artemether and artemisinin are relatively stable, while the RMSD value of artesunate shows periodic fluctuations between 0.15 nm and 0.2 nm. In addition, we observed that all ligands remained in the binding cavity during the whole simulation. The RMSD values of the ligands are all below 0.2 nm, indicating that they reached a stable state. The RMSD transition of Artesunate due to confomational switching owing to the rotation of the oxygen-carbonyl (O-C) bond (Figure S2).

### 3.5. RMSF Analysis

**Figure 5:**
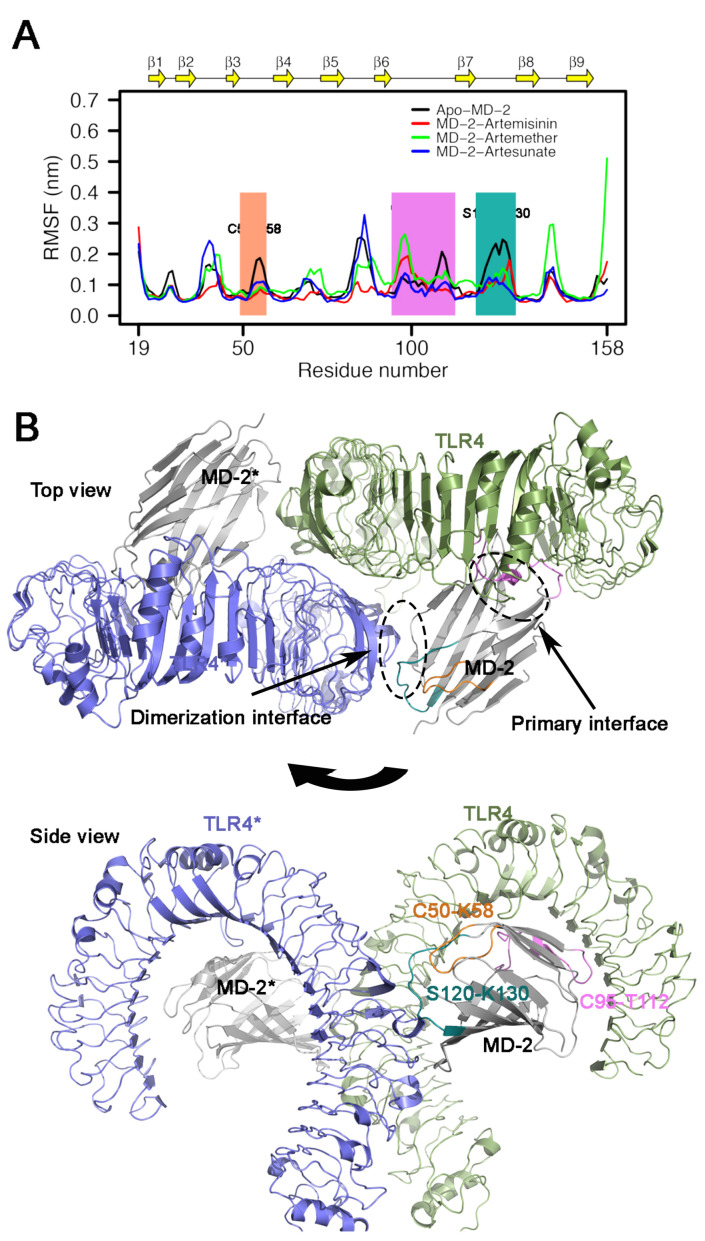
(**A**) Root mean square fluctuation in the last 50 ns of all systems with different colors. Significant fluctuations of loop regions are labeled. (**B**) Dimeric structure of the mTLR4/MD-2 complex. TLR4 and MD-2 are shown in green and gray, respectively, and their dimerization partners TLR4* and MD-2* are shown in blue and gray, respectively. Loop region C50–K58 is in orange, C95–T112 is in pink and S120–K130 is in green.

**Figure 6:**
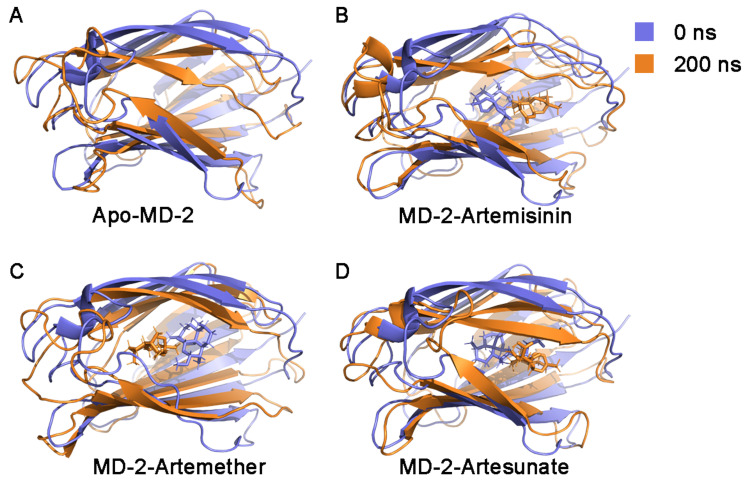
Structural superimposition of initial (0 ns) and final configurations (200 ns) of (**A**) Apo-MD-2, (**B**) MD-2-Arteminsinin complex, (**C**) MD-2-Artemether complex, and (**D**) MD-2-Artesunate complex. Initial conformations are colored in blue. Final conformations are colored in orange.

The root mean square fluctuation (RMSF) of backbone atoms of Apo-MD-2 and ligand binding MD-2 complexes were analyzed to measure the mobility of the MD-2 residues. As shown in Figure 5A, the large fluctuations of residues mainly occur in loops and in both termini regions, whereas relatively lower RMSF values are associated with β-strands for all simulations. The overall RMSF patterns are similar in the presence of ligand binding. In particular, ligand binding decreased the flexibility of loop between the β3 and β4 (residue C50–K58) region and between the β7 and β8 (residue S120–K130) region. Moreover, the fluctuation of RMSF between different ligands was also distinct, which may be related to the different binding sites of the ligands. The binding orientation of artemether, artesunate and artemisinin in the MD-2 pocket were altered significantly during the simulation (see Figure 6). As depicted in Figure 5B, the crystal structure reveals that the loop region β7–β8 is located at the dimerization interface of TLR4. This loop has been reported to play crucial roles in TLR4/MD-2 activation [34]. Specifically, amino acids Ile124 and Phe126 in this loop have been demonstrated experimentally through downstream signaling activation as mutation led to a reduced capacity to form dimer of TLR4/MD-2 complex [35,36]. In the presence of ligand, comparably low flexibility of Ile124 and Phe126 were observed. This observation may suggest that the binding of artemisinin and its derivatives to MD-2 reduces the conformational changes of the β7–β8 loop thereby inhibiting TLR4-MD-2 dimerization and preventing immune response. Although the loop region β3–β4 does not interact directly with TLR4, decreased fluctuations were observed upon ligand binding, suggesting that this region also had an influence on the dimerizaiton activity of TLR4-MD-2, which is consistent with previous TLR4-MD-2 simulations [37,38]. In contrast, the loop region bridging β6 and β7 (residue C95–T112) showed a increased fluctuations upon ligand binding, which is located at the primary contact interface between TLR4 and MD-2. As reported previously, the primary contact interface between TLR4 and MD-2 is formed before ligand binding [21,39,40]. This observation suggests that the binding of artemisinin and its derivatives to MD-2 reduces the TLR4-MD-2 stability.

### 3.6. Interactions between Inhibitors and MD-2

**Figure 7:**
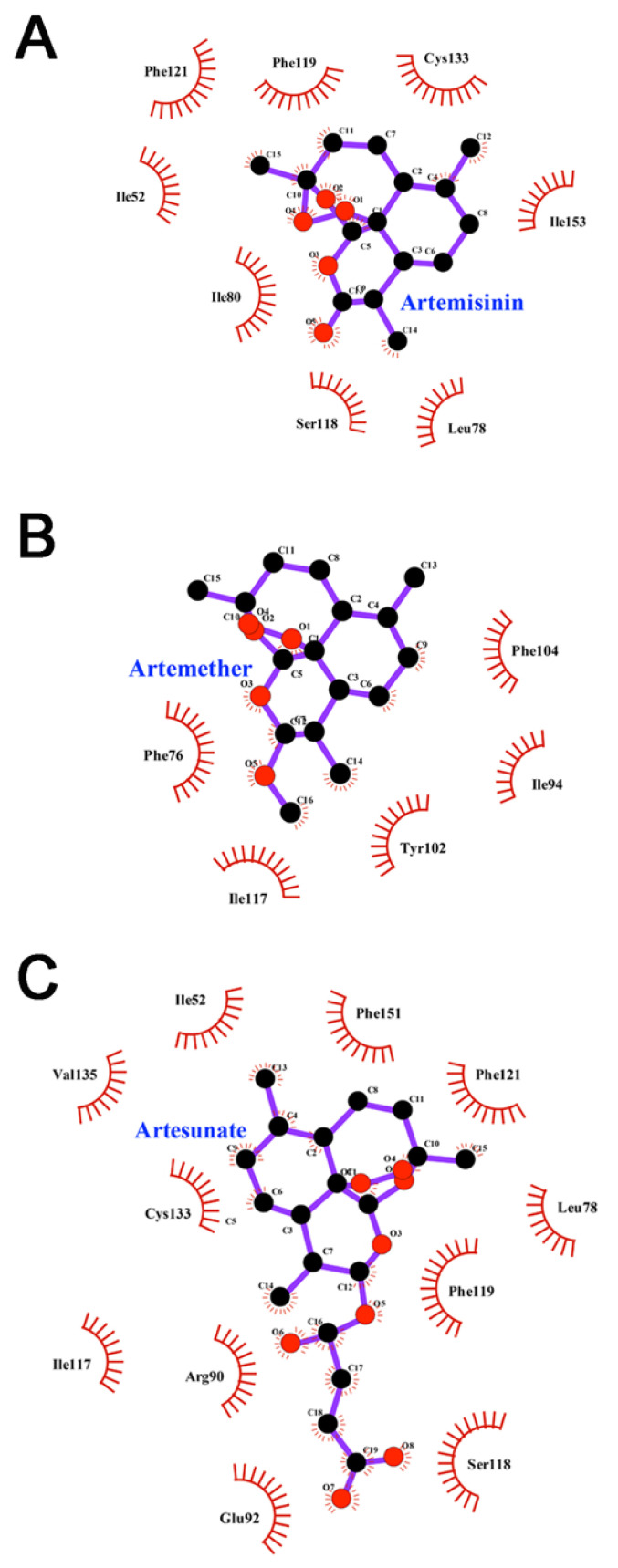
Interactions of MD-2 with (**A**) artemisinin, (**B**) artemether and (**C**) artesunate. The last frame of the MD simulation was used for these plot.

The Interactions between ligands and MD-2 were investigated using LigPlot [41]. As shown in Figure 7, artemisinin forms hydrophobic interactions with Ile52, Leu78, Ile80, Ser118, Phe119, Phe121, Cys133 and Ile153. Artemether forms hydrophobic interactions with Phe76, Ile94, Tyr102, Ile117 and Phe104. Artesunate forms hydrophobic interactions with Ile52, Leu78, Arg90, Glu92, Ile117, Ser118, Phe119, Phe121, Cys133, Val135 and Phe151. This indicated that all ligands interact effectively with MD-2 in the binding pocket and inhibit its activity mainly through hydrophobic interactions. Despite artesunate exhibits more hydrophobic interactions as compared with artemisinin and artemether, its inhibitory effect is lower than those of artemisinin and artemether. In addition, the main differences were observed from the interactions of residues Arg90 and Glu92 with ligands. These two residues were reported as the key residues to stabilize the complex by forming hydrogen bonds with ligand in the binding cavity of MD-2 [37,42], which may be related to the difference in ligand activity (see the next section).

**Figure 8:**
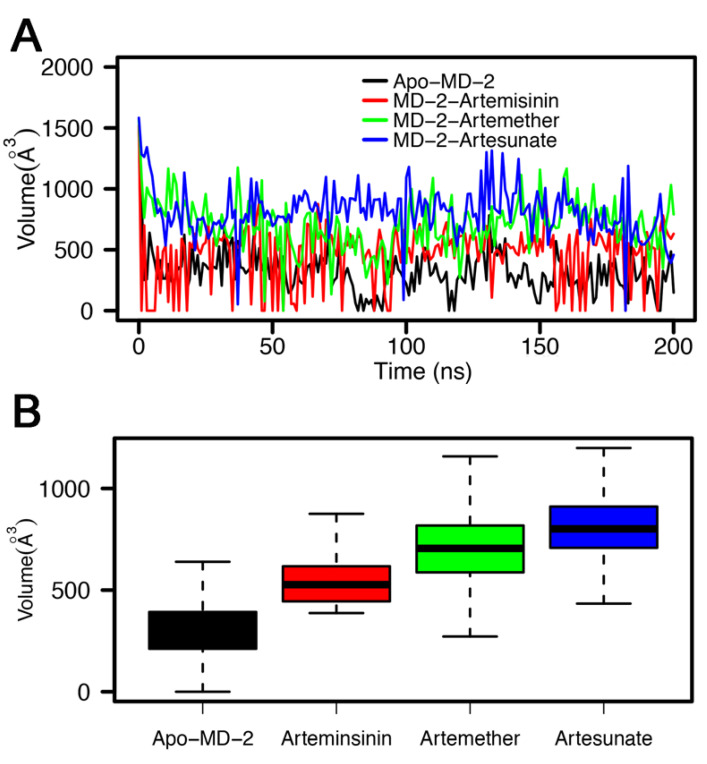
Ligand binding pocket volume (**A**) in line plot as a function of simulation time and (**B**) in barplot for Apo-MD-2, MD-2-Artemisinin, MD-2-Artemether and MD-2-Artesunate systems over the 200 ns simulation.

The overall secondary structure of these four systems was conserved except for the residues in the loop region (Figure S3). Despite no significant conformational change being found in the secondary structure, the binding of different ligands can lead to changes in the hydrophobic pocket, thus affecting the interactions between MD-2 and TLR4. Previous reports have also found that ligand properties determine the ’on-off’ state of MD-2 pockets. Previously, the cavity of MD-2 was reported as being malleable in both open and closed states and as undergoing a “clamshell-like” motion to adjust its volume and to precisely match the ligand’s proportions [43]. As expected, we observed that MD-2 rapidly closes in the absence of any ligand (Figure 6), the same phenomenon was also reported by Zhang et al. [15]. Interestingly, the MD-2 pocket undergoes plastic deformation also occurs in the presence of the ligand. Furthermore, the artemether-bound MD-2 shows less shrinkage. Thus, we calculated the volumes of ligand binding pockets throughout MD simulations in order to study changes in the pocket volume by trj_cavity plugin [44] for GROMACS with a dimension of 5 and a grid spacing of 1.4 Å. As shown in Figure 8, the volume of the pocket decreased rapidly in the first 10 ns and then fluctuated sharply during the simulation. The pocket volumes of different systems (Apo-MD-2, MD-2-Artemisinin, MD-2-Artemether and MD-2-Artesunate) are 314.93 ± 177.40 Å${}^{3}$, 464.64 ± 247.62 Å${}^{3}$, 703.79 ± 210.39 Å${}^{3}$ and 810.85 ± 205.33 Å${}^{3}$, respectively. In the absence of ligand binding, the cavity of Apo-MD-2 contracted sharply and its volume was minimal, which is consistent with the observed results. It is interesting to note that although the ligands have similar chemical structures, their volumes in the binding pocket are quite different. Artesunate exhibited the largest average pocket volume and the lowest anti-inflammatory activity, while artemether exhibited the best anti-inflammatory activity with moderate pocket volume among all ligands. In summary, our findings suggest that the ligand has a major impact on the plasticity of MD-2, thus regulating the activity of TLR4-MD-2.

### 3.7. MM-PBSA Binding Free Energy Calculations

Next, the quantitative estimation of the binding free energy of inhibitors against MD-2 was carried out using the MM/PBSA approach. The energy components and binding free energies of all complexes are listed in Table 1. The binding energy (ΔG) of MD-2-Artemisinin, MD-2-Artemether and MD-2-Artesunate complexes were −107.264 ± 0.793 kJ · mol ${}^{-1}$, −123.964 ± 0.766 kJ · mol ${}^{-1}$ and −112.196 ± 1.145 kJ · mol ${}^{-1}$, respectively. The negative value of ΔG indicates that all inhibitors favor staying in the MD-2 binding pocket. The van der Waals energy (ΔE${}_{vdw}$), the electrostatic interaction energy (ΔE${}_{elec}$), and the polar energy(ΔG${}_{polar}$) contribute negatively to the binging energy, whereas the nonpolar energy (ΔG${}_{non-polar}$) contributes positively to it. The ΔE${}_{vdw}$ has also been observed to be higher than ΔE${}_{elec}$ and ΔE${}_{non-polar}$ for all the complexes, indicating the ΔE${}_{vdw}$ is the major contributor to the binding free energy. Free energy calculations revealed that the artemether binds to MD-2 with an appreciable binding affinity rather than artemisinin and artesunate, finally, this results in the formation of a stable complex. This result is consistent with the experimental results.

**Figure 9:**
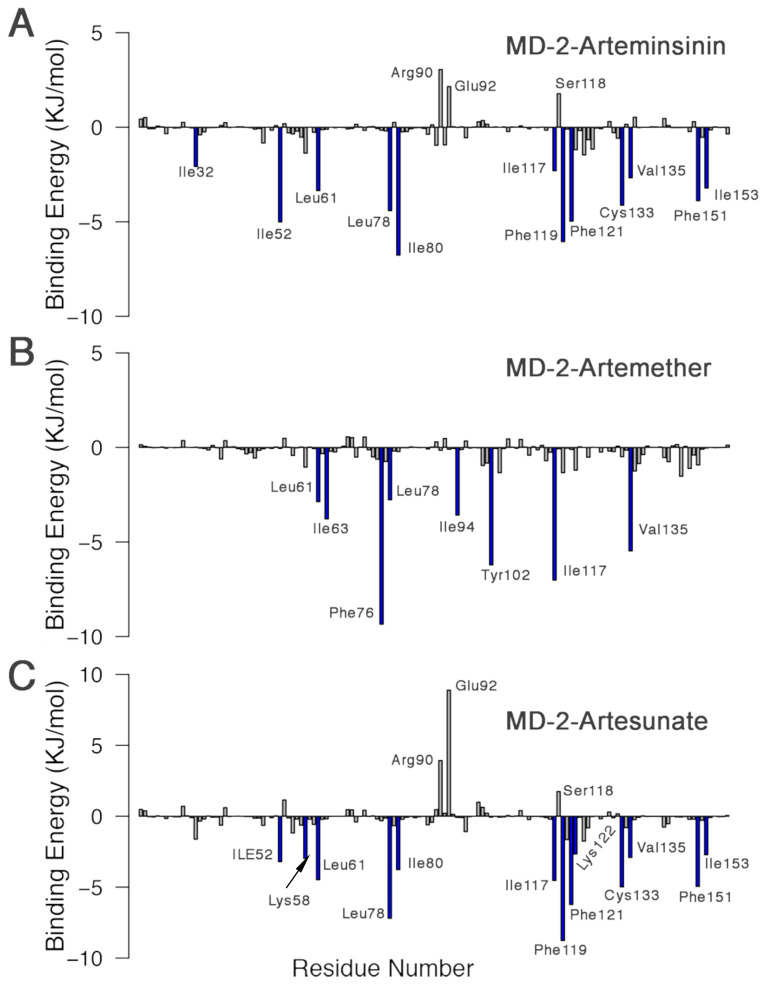
Per-residue decomposition of binding free energy contributions of (**A**) MD-2-Arteminsinin complex, (**B**) MD-2-Artemether complex, and (**C**) MD-2-Artesunate complex. Free energyies less than −2 KJ· mol ${}^{-1}$ are highlighted in blue.

Apart from the overall binding free energy, the contribution of residues to the binding energy in each system was determined. The amino acids that had significant impacts on the contribution of binding free energy (≤−2 KJ· mol ${}^{-1}$) were marked with blue bars. As shown in Figure 9, most residues contributed attractive MD-2-inhibitor interactions in all three systems, whereas Arg90, Glu92 and Ser118 contributed repulsive interactions, except for in the MD-2-Artemether complex. Residues Leu61, Ile63, Leu78, Arg90, Tyr102, Phe119, Ser120, Phe121, Lys122, Phe126, Gly123, Lys130, Cys133, and Phe151 have been reported as predictors of key residues in the interactions between MD-2 and nonlipid inhibitors such as JSH [45], taxanes [46], xanthohumol [47], nicotine, and cotinine [48]. Some of them can form hydrogen bonds with residues Arg90 and Glu92. In the case of artemisinin and its derivatives, the van der Waals energy was observed to contribute more to the binding energy. Figure 9 illustrates the major contributions of free energy coming from a few groups around Leu61, Leu78, and Ile117 in all systems. This indicates that residues Leu61, Leu78, and Ile117 are essential for artemisinin and its derivatives to stabilize the complexes.

## 4. Conclusions

Here, we combined experimental and computational methods to investigate the the binding patterns and key residues of artemisinin and its derivatives to MD-2. Artemisinin and its derivatives significantly attenuated LPS-induced increases in proinflammatory cytokine levels in BV2 microglial cells. The trend for anti-inflammatory activity is artemether > artemisinin > artesunate, and artemether had the highest inhibitory effect. To elucidate the binding modes of the MD-2-Artemisinin, MD-2-Artemether and MD-2-Artesunate complexes, we performed molecular docking. Furthermore, atomistic molecular dynamics simulations were performed to study the dynamics and stability of these complexes obtained from the docking study. Simulation results illustrate that hydrophobic interactions were critical in maintaining the binding mode of the ligands and MD-2. Furthermore, amino acids Leu61, Leu78, and Ile117 are indeed key residues that contribute to the binding free energy. The results of this study will be useful in the design and development of novel MD-2 inhibitors using an artemisinin-based template.

## Figures and Tables

**Table 1:** Binding energies of protein-ligand complexes obtained using g_mmpbsa.

Complex	ΔE${}_{\mathrm{vdw}}$	ΔE${}_{\mathrm{elec}}$	ΔG${}_{\mathrm{polar}}$	ΔG${}_{non-polar}$	ΔG${}_{\mathrm{binding}}$
MD-2-Artemisinin	−146.601 ± 0.665	−31.839 ± 1.113	87.203 ± 1.471	−16.017 ± 0.043	−107.264 ± 0.793
MD-2-Artemether	−136.635 ± 0.861	−16.698 ± 0.506	44.837 ± 0.822	−15.485 ± 0.056	−123.964 ± 0.766
MD-2-Artesunate	−181.365 ± 1.180	−108.712 ± 1.860	197.644 ± 2.708	−19.634 ± 0.058	−112.196 ± 1.145

ΔE${}_{vdw}$, ΔE${}_{elec}$, ΔG${}_{polar}$ and ΔG${}_{non-polar}$ are binding energy components of van der Waals, electrostatic, polar and nonpolar solvation energies, respectively. ΔG${}_{binding}$ is the total binding energy. The unit of energy is kJ · mol ${}^{-1}$.

## Data Availability

All data presented in this study are available on request from the corresponding author.

## Supplementary Materials

The following are available online: Figure S1: Intermolecular interactions in (A) MD-2-Artemether, (B) MD-2-Artemisinin and (C) MD-2-Artesunate from docking complex. Red circles shows the identical interacting amino acids in all systems. Figure S2: Structural superimposition of 10 structures of the Artesunate during the 200ns simulation (snapshots separated by 20 ns) and chemical structures of Artesunate. Initial (0 ns) configuration is colored in blue, switching bond is colored in red., Figure S3: Secondary structure of MD-2 in (A) Apo-MD-2, (B) MD-2-Artemether, (C) MD-2-Artemisinin and (D) MD-2- Artesunate complex.

## Abbreviations

The following abbreviations are used in this manuscript:

TLR4	Toll-like receptor 4
LPS	lipopolysaccharide
MD-2	myeloid differentiation factor 2
PAMPs	pathogen-associated molecular patterns
RMSD	Root mean square deviation
RMSF	Root mean square fluctuation
CCK-8	Cell Counting Kit 8
