# Supplementary material for: Molecular Basis of Artemisinin Derivatives Inhibition of Myeloid Differentiation Protein 2 by Combined in Silico and Experimental Study
Source: Molecules. 2021 Sep 20;26(18):5698. doi: 10.3390/molecules26185698 (PMC8469597; doi:10.3390/molecules26185698)
Supplement: Supplementary file 1 [file molecules-26-05698-s001.zip › molecules-1368386-supplementary.pdf]

Article

# Molecular basis of Artemisinin derivatives inhibition of Myeloid differentiation protein 2 by combined in silico and experimental study

**Sennan Qiao [1], Hansi Zhang [2] , Fei Sun [1,*] and Zhenyan Jiang [1,*]**

[1] School of Pharmaceutical Sciences, Jilin University, Changchun,130021, China
[2] College of Basic Medical Sciences, Jilin University, Changchun,130021, China
* Correspondence: sunfei@jlu.edu.cn; zy_jlu2016@163.com;

**Keywords:** Molecular dynamics simulation; myeloid differentiation factor 2; artemisinin; anti-inflammatory ;Binding free energy

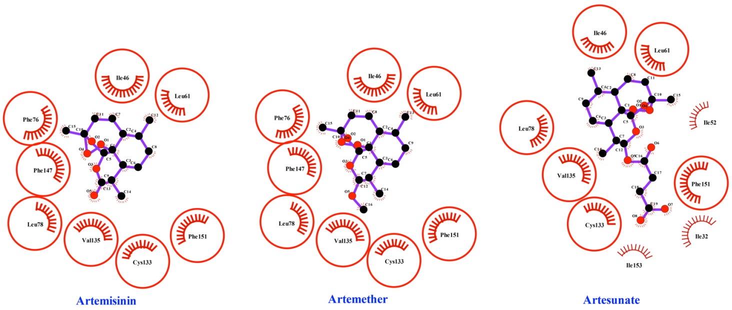


**Figure S1.** Intermolecular interactions in (A) MD-2-Artemether, (B) MD-2-Artemisinin and (C) MD-2-Artesunate from docking complex. Red circles shows the identical interacting amino acids in all systems.

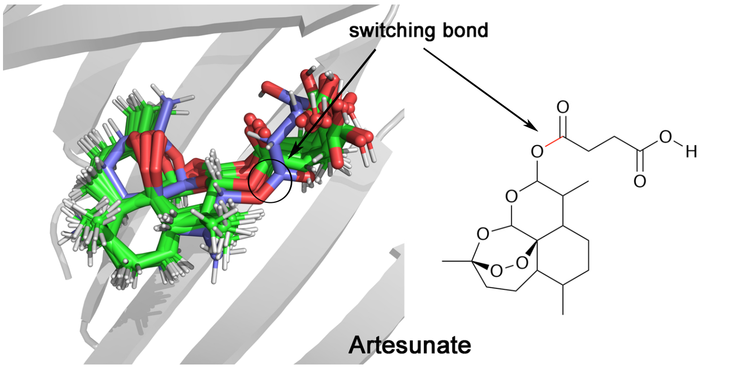


**Figure S2.** Structural superimposition of 10 structures of the Artesunate during the 200 ns simulation (snapshots separated by 20 ns) and chemical structures of Artesunate. Initial (0 ns) configuration is colored in blue, switching bond is colored in red.

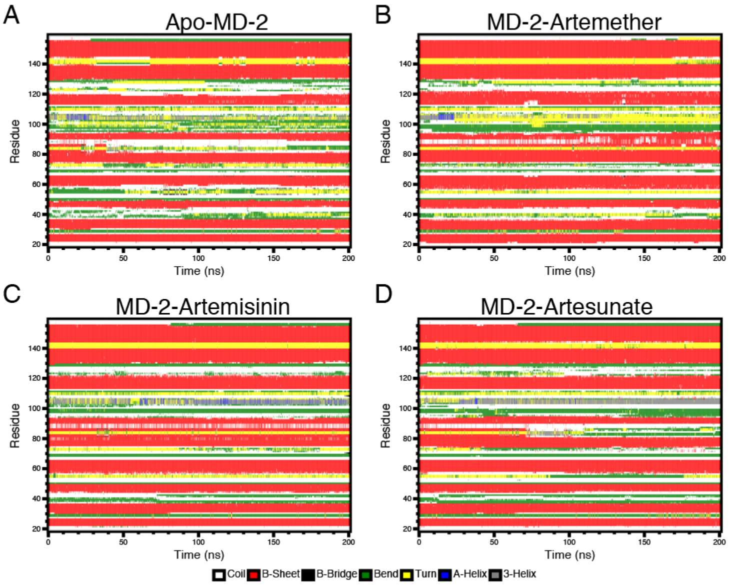


**Figure S3.** Secondary structure of MD-2 in (A) Apo-MD-2, (B) MD-2-Artemether, (C) MD-2-Artemisinin and (D) MD-2-Artesunate complex.
